# A Throughput and Energy Efficiency Scheme for Unlicensed Massive Machine Type Communications †

**DOI:** 10.3390/s20082357

**Published:** 2020-04-21

**Authors:** Iran Ramezanipour, Hirley Alves, Pedro H. J. Nardelli, Ari Pouttu

**Affiliations:** 1 Centre for Wireless Communications, University of Oulu, 90570 Oulu, Finland; hirley.alves@oulu.fi (H.A.); ari.pouttu@oulu.fi (A.P.); 2 School of Energy Systems, LUT University, 53850 Lappeenranta, Finland; pedro.nardelli@lut.fi

**Keywords:** energy efficiency, massive machine type communications, poisson point process, spectrum sharing, unlicensed spectrum access

## Abstract

In this paper, the throughput and energy efficiency of an unlicensed machine type communications network is studied. If an outage event happens in the network, there is a possibility for packet retransmission in order to obtain a lower error probability. The concept of spectrum sharing is used here for modeling the network, which allows the two types of licensed and unlicensed users to share the same uplink channel allocated to the licensed users. However, it is done in a way that no harm is done to the licensed nodes’ transmission for sharing the same channel with the unlicensed users, while licensed nodes’ transmission causes interference on the unlicensed network. Poisson point process is used here to model the location of the nodes and the effect of interference on the network. We study how different factors such as the number of retransmissions, SIR threshold and outage can affect the throughput and energy efficiency of the network. Throughput and energy efficiency are also both studied in constrained optimization problems where the constraints are the SIR threshold and the number of retransmission attempts. We also show why it is important to use limited transmissions and what are the benefits.

## 1. Introduction

Internet of things has revolutionized the way communication works and is slowly becoming a part of our daily lives. Many applications are currently becoming IoT based, from remotely controlling your house to different processes in an industrial setting [1]. Billions of devices are expected to join the Internet by the year 2020, which while providing a big economic impact, will also create new challenges such as the availability of spectrum resources [2,3]. This makes finding ways to efficiently use the spectrum more valuable than ever. Machine-type communications (MTC) is a non-human centric concept introduced under the umbrella of IoT for the future communications technology, 5G, which can support a high number of connectivity in the network and can provide different quality of services [4].

MTC can be divided into three categories based on the expected properties, (i) enhanced mobile broadband (eMBB); which should be able to provide connectivity with high peak rates in addition to moderate rates for the cell-edge users; (ii) ultra-reliable MTC (uMTC), which focuses on making ultra reliable and low latency connections in the networks possible and (iii) massive machine type communication (mMTC), whose main goal is to provide massive connectivity for a large number of nodes (in the order of 10 times higher than the current number of connected devices) with different quality of service (QoS) [5,6]. A mMTC network usually consists of billions of low-complexity low-power machine-type devices as nodes. A good example of this type of networks are smart grids where the data from a very large number of nodes (smart meters) needs to be collected [7,8,9]. Industrial control is also another application of mMTC. In both of these examples, the reliability level of the network needs to be high, and it also should be able to handle critical situations [10]. A very good recent study on why 5G and MTC are needed in order to achieve the expected performance in smart grids, specifically with regards to grid protection and control is done in [11]. Motivated by this, in our work, we focus on throughput optimization and energy efficiency in a mMTC network in the presence of retransmissions.

The spectrum resources are limited, and hence, the availability of spectrum is a never ending challenge for wireless communications. Considering that mMTC is going to connect billions of devices together, this notion is becoming even more challenging in the upcoming 5G networks. Thus, studying different ways to efficiently use the available spectrum is very important [8]. This is specifically important in the future 5G technology, specially mMTC, since suitable spectrum resources below 6 GHz are limited. Moreover, it is not really possible to use mmWave for mMTC applications since they are limited in terms of resources and are mostly implemented in locations where we could face propagation problems, such as indoor or underground locations [12].

Keeping all these in mind, spectrum sharing can provide useful tools which can help mMTC networks to use the spectrum more efficiently [13,14,15,16]. One of these methods is the unlicensed spectrum access, which is a suitable option for low-power IoT-based networks and is also the spectrum access method used in this paper. It should be noted that while the studied model here is based on the concept of cognitive radios and spectrum sharing, the focus is not on design and issues related to these networks but rather on evaluating different performance metrics important to these kinds of networks. Our analysis is motivated by the aforementioned lack of spectrum resources and better ways to use these limited resources and increase the spectral efficiency of the network.

A valuable study has been done in [17] where the authors study the energy grid as an important case of mMTC and propose and evaluate using the LTE network as a communication tool in these networks. Moreover, in [18,19], the previously mentioned unlicensed spectrum access model is studied, where the unlicensed users use the licensed nodes uplink channel too. In there, they show that the positions of the unlicensed users are fixed, which makes it reasonable to use highly directional antennas and limited transmit power in the unlicensed network in order to avoid interference from this network affecting the licensed network. However, this does not prevent the licensed nodes from causing interference on the unlicensed users. Since these works are limited to smart grids applications, later on we expanded these works in [20], by following the work done in [21,22], to make the model more generalized and compatible with other wireless networks. In [23], authors propose a spectrum sharing method for a MTC network in LTE with large number of nodes where simultaneous transmissions are possible. In this model, dynamic spectrum access is possible for a dense network with low data rates. The advantage of this model is that there is no need for signaling procedures that are going to need channel reservation. Moreover, in [24], authors introduce interesting new schemes which make implementing cognitive radio and spectrum sharing methods in smart gird applications easier. They also study a number of routing protocols and interference mitigation schemes related to smart grids. A valuable study has been done in [25] regarding the resource allocation in different spectrum sharing networks such as SINR-based, transmission power-based, and centralized and distributed methods of decision making. In [26], Liu et al. propose a novel multichannel IoT scheme for dynamic spectrum sharing in 5G networks. In their work, an IoT node is able to do two different types of communications at the same time, both 5G and IoT communications. This is done by allocating different sub-channels for these two communication modes.

All the above mentioned works have done great works with regards to addressing spectrum sharing in IoT and machine type communications; however, they do not aim at optimizing the performance of the system while having different reliability constraints, which is one of the main pillars of future 5G. In this paper, we expand one of the scenarios in our previously published work [27] and follow the same model as in [18,20,28] to prove that the approximation used in those papers for the average number of retransmission attempts is in fact a tight approximation. We show why it is worth to use limited number of retransmission, and optimize the throughput as a function of the SIR threshold and number of retransmissions. Moreover, we also study the energy efficiency of the proposed model in this paper. Energy efficiency is one of the most important issues that needs to be considered in wireless networks, specifically in ultra dense networks [29]. As was previously mentioned, mMTC networks are designed to support massive connectivity between billions of sensors and IoT devices with minimum human interactions [5], most of which are low powered. Most of theses devices are battery supplied, and hence, they have a limited energy supply (wireless sensor networks for instance). It also happens often that these mMTC networks are deployed in critical or hard to access locations, which makes changing the batteries and renewing the energy resources very difficult [30,31,32]. All these reinforce the importance of the energy efficiency in mMTC in general.

Valuable works have been done in the field of energy efficiency. In [33], authors study different challenges and metrics with regards to reducing the total power consumption of the network while in [34,35], maximizing the energy efficiency by optimizing the packet size and constrained by an outage threshold in non-cooperative and cooperative wireless networks is studied respectively. In [36], a new scheduling algorithm based on frame aggregation is proposed in order to achieve energy efficiency in IEEE 802.11n wireless networks. In terms of the physical properties of the battery equipped machine type devices, two interesting medium access control protocol and power scheduling schemes are proposed in [37,38] in order ro preserve battery life. Moreover in [39], Takeshi Kitahara et al. introduce a data transmission control method based on the well-known electrochemical characteristics of batteries, which makes increasing the discharge capacity possible.

While the above mentioned works are interesting and valuable, none of them really addresses the massive connectivity issue and how the energy efficiency needs to be handled in a mMTC network. In [40], the authors investigate access control algorithms for machine type nodes, which can reduce the energy consumption in the uplink channel. There, the machine type nodes access to the base station is maximized by means of grouping and coordinator selection. However, in this paper, we evaluate the energy efficiency of an unlicensed mMTC network and optimize the energy efficiency constrained by an outage threshold and maximum number of retransmissions. We also show the effect of different network parameters such as network density and the SIR threshold on the behavior of the energy efficiency.

The following are the main contributions of this paper.

- We show how tight is the approximation used for the average number of retransmission attempts in our other work and the desired range that the approximation is valid for.
- The maximum number of allowed retransmissions and the SIR threshold that leads to the maximum link throughput is studied.
- The optimal throughput in the sense of spectral efficiency and the energy efficiency of the optimal throughput are also studied. Moreover, we show why it is important to have limited transmissions in the network and why it is beneficial to use our proposed optimal throughput model.
- The effect of different network properties such as network density and the SIR threshold on the optimal throughput and the energy efficiency of the network are also studied.

The rest of the paper is divided as follows. Section 2 introduces the network model and how the Poisson point process is used to model the network and carry out the analysis, while Section 3 details the throughput optimization analysis. In Section 4, we study the energy efficiency and the energy efficiency of the optimal throughput. Section 5 presents the numerical results while Section 6 concludes this paper. Table 1 provides a list of functions and symbols used in this paper.

## 2. System Model

**Figure 1:**
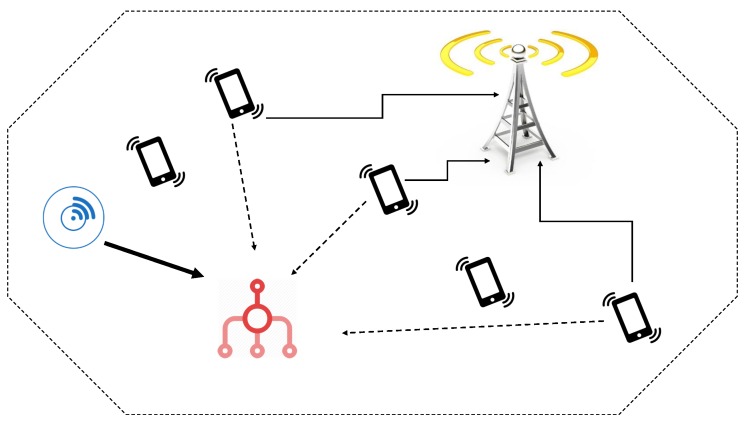
An illustration of the dynamic spectrum access scenario, where licensed and unlicensed users share the up-link channel. The unlicensed transmitter is depicted by the smart meter, the aggregator (unlicensed receiver) by the red aggregator, the handsets are the mobile licensed nodes (interferers to the aggregator) and the big antenna is the cellular base-station. As the smart meter uses directional antennas with limited transmit power (bold arrow), its interference towards the base-station can be ignored. The thin black arrows represent the licensed nodes’ desired signal, while the dotted ones represent their interference towards the aggregator.

In order to do the throughput and energy efficiency analysis in this paper, we use a dynamic spectrum access network model, shown in Figure 1. The two types of nodes in this model, which are licensed and unlicensed nodes. These nodes share the same frequency band which is used by the licensed nodes in the uplink channel. This sharing is done in a way that the unlicensed users do not cause any interference on the licensed nodes. In this model, the licensed link is the one between the mobile users and the cellular base stations while the unlicensed link is what is used by the sensors to communicate with their corresponding aggregators. The unlicensed users are considered to be static, which would make use of directional antennas with restricted radiation pattern; hence, the orientation errors could be avoided.

We assume here the ideal case, where the beam can be modeled as a straight line. Although this is a strong assumption, it can be seen as the theoretical limit of this scheme. A more realistic beam with orientation errors has been studied, for example, in [41] where Wildman et al. study different trade offs regarding the network throughput and transmission capacity with changes in the antenna beamwidth. In that paper, the authors study an ad hoc network implemented using bipolar Poisson point process and use stochastic geometry in order to analyze the optimal beamwidths that maximize different throughput-based metrics, including the orientation errors. They have shown that in case of having a fixed transmit power, decreasing the beamwidth will increase the antennas main beam gain, which in its turn will result in lower interference from the interfering nodes on the receiver. In such situation, aligning the transmitter and receiver appropriately would be more challenging; however, when they are aligned, it will result in an increase in the desired signal strength.

In this paper, we do not consider how the link between sensor and aggregator is formed since the unlicensed network has a fixed topology. In this model, only the typical link in the unlicensed network suffers from the interference caused by the uplink licensed network. The licensed network, on the other hand, is highly mobile in the sense that the topology of active transmitters change at every transmission attempt of the unlicensed network. Hence, we have the following assumptions to evaluate our scenario:

- Unlicensed network: We assume only a reference link, which is fixed and associated with a specific gateway/base-station/aggregator.
- Licensed network: This is not evaluated in this work. We only assume that at every transmission attempt by the unlicensed reference link, there is a random number of active users in the licensed network with a random relative position in relation to the aggregator node (thus, we can model the positions of the active transmitters of the licensed network in respect to the reference aggregator of the unlicensed as a Poisson Point Process).

The power used by the unlicensed users for their transmission is limited. This limitation which is either imposed on them from the licensed network or comes from sensors own properties, restricts the maximum range that the unlicensed network transmitted signal can reach. This means that the unlicensed users radiation patterns can be considered as a line segment in which the starting points are these nodes, and the end point is determined by the power constraint. Moreover, it is assumed that in this model, there are no packet collisions between the transmitted packets by the sensors to the same aggregator. This is justifiable since the packet size in these transmissions is assumed to be small and the unlicensed network can take advantage of the multiple access solutions. Another important aspect that should be considered in this model is that before the unlicensed users use the licensed nodes channel, they first evaluate the channel usage and then do their transmission if they sense the channel is free; otherwise, they have to postpone their transmission or use another frequency band [42,43].

Considering the above explanations, we can see that the interference in this model can stem from the following different sources, (i) from the mobile users to aggregators, (ii) from sensors to cellular base stations and (iii) from sensors to aggregators that they are not communicating with. Taking into account the aforementioned system model assumptions, it is possible to conclude that if the licensed and unlicensed nodes are implemented in explicit positions, it makes it possible to eliminate the interferences in cases (ii) and (iii).

Following [18,27], we assume that (iii) can be eliminated by a specific access policy since the links between sensors and aggregator are deterministic (each sensor is assumed to be connected with one specific and known aggregator). Therefore, the aggregator can allocate orthogonal channels or different time slots to each sensor. Besides, due to the narrow beam and limited transmit power, the interference from sensors related to different aggregators can be neglected. Even if it is assumed that the nodes are implemented in random locations, the probability of having the base station or aggregator in the same line segment as the unlicensed users signal is close to zero [18,20,28]. As follows, the only source of interference in this model is then (i). In order to be able to evaluate the impact of this interference on the system performance, we need to be able to have a good understanding of the uncertainty of the licensed nodes’ locations. Hence, a Poisson point process Φ is used to model the interfering nodes distributed over an infinite two-dimensional plane where λ > 0 (average number of nodes per m^2^) denotes the spatial density. This model is also elaborated in detail in [20,44].

For modeling the wireless channel in this paper, we consider distance dependent path-loss and quasi-static fading model. Here, $r_{i}$ represents the distance between the reference receiver and the *i*-th interfering node. Based on Slivnyak theorem [44], the reference receiver being located arbitrarily at the origin means that the receiver’s position is at the center of the Euclidean space. Having this location as a fixed point makes locating the surrounding elements easier [27,44]. More detailed explanation about using this tool in modeling mMTC networks and why it is useful can be found in [45,46]. The channel gain between the reference receiver and the *i*-th interfering node is shown as $g_{i}$. If we consider $P_{T_{x}}$ to be the transmit power and α > 2, the path-loss exponent, then the power received at the reference receiver is equivalent to $P_{T_{x}}g_{i}r_{i}^{-\alpha }$. With these in hand, the signal to interference ratio at the reference receiver SIR_0_ Signal-to-interference-plus-noise ratio (SINR) is defind as $\mathrm{SINR}=\frac{S}{N\;+\;I}$ where S denotes the desired signal power and N and I the noise power and interference power, respectively; however, in interference limited scenarios such as the one studied in this paper, where noise can be neglected, the SINR is reduced to signal-to-interference ratio (SIR) [44].) is then defined as:

(1) $${\mathrm{SIR}}_{0}=\frac{P_{s}g_{0}r_{0}^{-\alpha }}{P_{p}\;\sum_{i\in \Phi }g_{i}r_{i}^{-\alpha }},$$

in which, $P_{p}$ denotes the licensed nodes transmit power and $P_{s}$ is the transmit power used by the unlicensed users for their transmissions. (Note that noise is not considered in this analysis, even if it is considered, adding noise would not have much effect on the output as also stated in [47].) The data rate in this model is defined as log(1+β) in bits/s/Hz. This is justifiable since the reference link takes advantage of point-to-point Gaussian codes and interference-as-noise decoding rules [20,48,49]. This spectral efficiency can only be achieved if the SIR is greater than a given threshold, which here is defined as β, i.e., SIR>β. An outage event happens if a transmitted message is not successfully decoded at the receiver side, meaning that SIR≤β. The probability of the system being in outage is $P_{\mathrm{out}}=\mathrm{Pr}\left[\mathrm{SIR}<\beta \right]$. In this model, there is possibility of retransmission in case an outage event occurs. There can be up to *m* retransmissions in the network; hence, if the message is not successfully decoded by the receiver after 1+m transmission attempts (first transmission plus *m* retransmissions), it is then dropped [21], which will result in packet loss. Here, $P_{\mathrm{suc}}$ is used to refer to the probability of having a successfully decoded message and is $P_{\mathrm{suc}}=1-P_{\mathrm{out}}^{1+m}$.

In order to compute $P_{\mathrm{out}}$, the previously mentioned channel gains *g* are considered to be quasi-static (squared envelopes), which are also independent and identically distributed exponential random variables (Rayleigh fading) with mean 1 [41]. In this model, the licensed nodes, which are also the source of interference, are not static but rather highly dynamic. Therefore, we consider that their locations with respect to the reference receiver are constantly changing during each transmission. Considering a Poisson point process Φ, it is possible to characterize the signal-to-interference ratio at the reference link SIR_0_ as in [20]. Then, the outage probability $P_{\mathrm{out}}=\mathrm{Pr}\left[{\mathrm{SIR}}_{0}\leq \beta \right]$ for each transmission attempt is as presented below (we assume that the distance $r_{o}$ is fixed (but arbitrary) due to the fixed topology we assumed for the unlicensed network. However, this assumption could be easily relaxed following Nardelli et al. work [50] by taking the expected value of the performance metric under investigation in relation to the distribution of $r_{o}$.) where $k=\pi r_{0}^{2}\Gamma \left(1-\frac{2}{\alpha }\right)\Gamma \left(1+\frac{2}{\alpha }\right)$ [20].

(2) $$P_{\mathrm{out}}=1-e^{-k\lambda \beta^{2/\alpha }}.$$

We are now able to evaluate the link throughput *T* in the reference link as:

(3) $$T=\frac{\log(1+\beta )}{1+\bar{m}}\left(1-P_{\mathrm{out}}^{1+m}\right),$$

It should be noted that in this throughput equation, *m* shows the retransmissions attempts whereas $1+\bar{m}$ is the average number of transmissions for a successful transmission. Further details shall be seen in Section 3. As stated above, $\left(1-P_{\mathrm{out}}^{1+m}\right)$ is the probability of having a successful transmission.

## 3. Throughput Optimization

### 3.1. Constrained Optimization

In this section we evaluate the optimal link throughput in the sense of spectral efficiency. We consider a constrained throughput optimization problem where the constraint is a maximum acceptable error rate imposed by the application at hand. This means that the quality requirement of this network is determined by how often a message is eventually dropped after all retransmission attempts. Considering (3), the optimization problem is defined as below.

(4) $$\begin{array}{cccc} \; & \max_{\left(\beta ,m\right)}\; & \; & \frac{\log(1+\beta )}{1+\bar{m}}\left(1-P_{\mathrm{out}}^{1+m}\right)\\ \; & \mathrm{subject}\;\mathrm{to}\; & \; & P_{\mathrm{out}}^{1+m}\leq \epsilon , \end{array}.$$

where ϵ represents the aforementioned quality requirement. In this equation, the SIR threshold β > 0 and the number of allowed retransmissions m∈N are the design variables.

**Lemma** **1.** The throughput T in (3) is a function of the variables m>0 and β>0, i.e., T=f(β,m). The function f is then concave with respect to β if $\frac{\partial^{2}T}{\partial \beta^{2}}<0$. After that, we can calculate $\beta^{*}$, which is the value of the SIR threshold that maximizes the link throughput.

(5) $$\beta^{*}={\left(-\frac{1}{k\lambda }\log\left(1-\epsilon^{\frac{1}{m+1}}\right)\right)}^{\frac{\alpha }{2}}.$$

**Proof.**  As *m* and β are strictly positive variables, and function *T* is twice differentiable in terms of β, then *T* is concave if and only if $\frac{\partial^{2}T}{\partial \beta^{2}}<0$. Based on our calculations, we can see below that the second derivative of the throughput equation is in fact negative with respect to β. Hence, in the region $\frac{\partial^{2}T}{\partial \beta^{2}}<0$, *T* is concave.

(6) $$\frac{\partial^{2}T}{\partial \beta^{2}}=-\frac{\left(-\epsilon +1\right)\left(-\epsilon^{\frac{1}{m+1}}+1\right)\left(-\epsilon^{m+1}+1\right)}{{\left(\beta +1\right)}^{2}\left(-\epsilon \left(-\epsilon^{\frac{1}{m+1}}+1\right)\left(m+1\right)-\epsilon +1\right)},$$

Knowing this, we can now calculate $\beta^{*}$. To do so, we follow the same steps as in ([18], Prop.1) where $\beta^{*}$ is the highest value that satisfies the inequality $1-P_{suc}\leq \epsilon$, thus $\beta^{*}$, which is presented as (5).

With this result at hand, we move forward as follows. The constraint in the optimization problem is $P_{\mathrm{out}}^{1+m}\leq \epsilon$. By considering the equality part $P_{\mathrm{out}}^{1+m}=\epsilon$ in addition to ([51], Â§17), we reach the below equation for the average number of retransmission attempts $1+\bar{m}$:

(7) $$1+\bar{m}=\frac{-\epsilon \left(-\epsilon^{\frac{1}{m+1}}+1\right)\left(m+1\right)-\epsilon +1}{\left(-\epsilon +1\right)\left(-\epsilon^{\frac{1}{m+1}}+1\right)}.$$

By inserting (2) and (7) into (3), we reach the following as the throughput equation.

(8) $$T=\log\left(\beta +1\right)\frac{\left(-\epsilon +1\right)\left(-\epsilon^{\frac{1}{m+1}}+1\right)\left(-\epsilon^{m+1}+1\right)}{-\epsilon \left(-\epsilon^{\frac{1}{m+1}}+1\right)\left(m+1\right)-\epsilon +1}.$$

 □

(9) $$\begin{array}{cc} m^{*}=\max_{m\in N} & \;\;\frac{\alpha \epsilon^{\frac{1}{m+1}}{\left(-\frac{1}{k}\log\left(-\epsilon^{\frac{1}{m+1}}+1\right)\right)}^{\frac{\alpha }{2}}\left(-\epsilon +1\right)\left(-\epsilon^{m+1}+1\right)\log\left(\epsilon \right)}{2{\left(m+1\right)}^{2}\left({\left(-\frac{1}{k}\log\left(-\epsilon^{\frac{1}{m+1}}+1\right)\right)}^{\frac{\alpha }{2}}+1\right)\left(-\epsilon \left(-\epsilon^{\frac{1}{m+1}}+1\right)\left(m+1\right)-\epsilon +1\right)\log\left(-\epsilon^{\frac{1}{m+1}}+1\right)}\\ \; & +\frac{\epsilon^{\frac{1}{m+1}}\left(-\epsilon +1\right)\left(-\epsilon^{m+1}+1\right)\log\left(\epsilon \right)\log\left({\left(-\frac{1}{k}\log\left(-\epsilon^{\frac{1}{m+1}}+1\right)\right)}^{\frac{\alpha }{2}}+1\right)}{{\left(m+1\right)}^{2}\left(-\epsilon \left(-\epsilon^{\frac{1}{m+1}}+1\right)\left(m+1\right)-\epsilon +1\right)}\\ \; & -\frac{\epsilon^{m+1}\log\left(\epsilon \right)\log\left({\left(-\frac{1}{k}\log\left(-\epsilon^{\frac{1}{m+1}}+1\right)\right)}^{\frac{\alpha }{2}}+1\right)}{-\epsilon \left(-\epsilon^{\frac{1}{m+1}}+1\right)\left(m+1\right)-\epsilon +1}\left(-\epsilon +1\right)\left(-\epsilon^{\frac{1}{m+1}}+1\right)\\ \; & +\frac{\log\left({\left(-\frac{1}{k}\log\left(-\epsilon^{\frac{1}{m+1}}+1\right)\right)}^{\frac{\alpha }{2}}+1\right)}{{\left(-\epsilon \left(-\epsilon^{\frac{1}{m+1}}+1\right)\left(m+1\right)-\epsilon +1\right)}^{2}}\left(-\epsilon +1\right)\left(-\epsilon^{\frac{1}{m+1}}+1\right)\left(-\epsilon^{m+1}+1\right)\\ \; & \times \left(\frac{\epsilon \epsilon^{\frac{1}{m+1}}}{m+1}\log\left(\epsilon \right)+\epsilon \left(-\epsilon^{\frac{1}{m+1}}+1\right)\right) \end{array}$$

**Proposition** **1.** The maximum number of allowed retransmissions $m^{*}$ that maximizes the link throughput is then given by (9).

**Proof.**  By taking into account the $\beta^{*}$ from (5) and (8), we can find the optimal maximum number of retransmissions ($m^{*}$) for the throughput. Hence, the optimal throughput $T^{*}$ in terms of both *m* and β is then given by the value of *m* that maximizes the throughput, which is achieved by (9). It should be noted that the maximum number of retransmissions *m* is a natural number that is usually small and, therefore, (9) is easy to evaluate. □

### 3.2. Extreme Cases

In this section, we evaluate the two extreme cases in terms of maximum number of transmissions and their effect on the throughput. To do so, we consider having no transmission m=0 and having a very high number of retransmissions m→∞ in the network. Considering the zero transmission case (A detailed analysis of the model studied in this paper while m=0 can be found in our previous work [18].), we reach the following for the throughput:

(10) $$\begin{array}{cc} T & =\log\left(1+\beta \right)\left(1-\epsilon^{\frac{1}{m+1}}\right)\\ \; & =\log\left(1+\beta \right)\left(1-P_{\mathrm{out}}\right)\\ \; & =\log\left(1+\beta \right)e^{-k\lambda \beta^{2/\alpha }}. \end{array}$$

Moving on to the m→∞ case, by replacing $\epsilon =P_{\mathrm{out}}^{\left(1+m\right)}$ in (7), we reach the below equation for the average number of retransmissions for the extreme cases ($1+{\bar{m}}_{e}$):

(11) $$1+{\bar{m}}_{e}=\frac{P_{\mathrm{out}}^{\left(1+m\right)}\left(1+m\right)\left(P_{\mathrm{out}}-1\right)-P_{\mathrm{out}}^{\left(1+m\right)}+1}{\left(P_{\mathrm{out}}-1\right)\left(P_{\mathrm{out}}^{\left(1+m\right)}-1\right)},$$

now by inserting (11) in (3), we can achieve the throughput equation in this case. After some mathematical manipulation, it is proven that while m→∞, we reach the same throughput equation as in (10). Hence, for both extreme cases of m=0 and m→∞, the system behavior remains the same in terms of throughput. We later use these results in the numerical results section of this paper and discuss what they show and why they are important. More details about these two extreme cases can also be found in [52].

### 3.3. Error Analysis

In our previous work, we can see that in ([20], Lemma 2), we use an approximation of (7) (which is also shown in this paper as (12)) presented in this analysis in order to calculate the average number of retransmissions in the throughput optimization problem in [20]. In this section we aim to prove that this previously used approximation is in fact a tight and good approximation, and the error in calculations is low when using the approximated expression. It is important to remember that this approximation is suitable when the number of transmissions is not large and also ϵ < 40%, which is the case in most of practical communications anyway since having a very large *m* is not efficient, and 40% is already a quite a loose error threshold for most systems.

**Figure 2:**
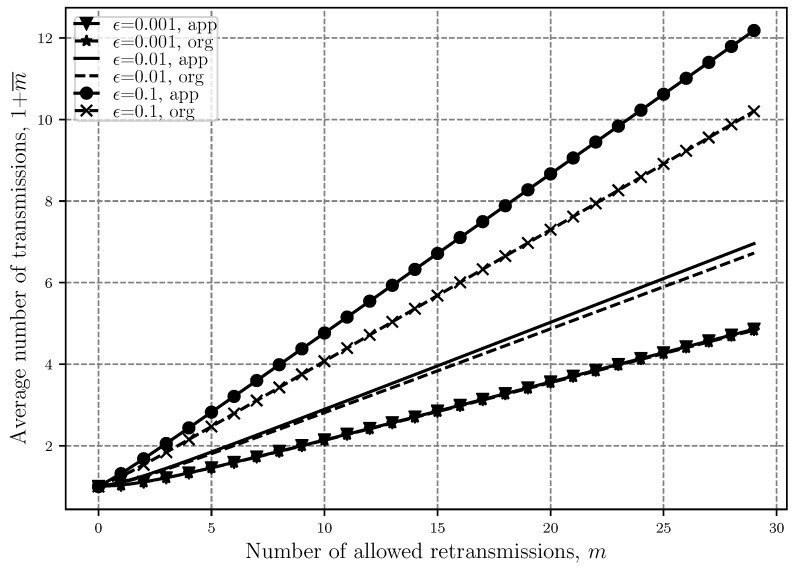
Comparison between the approximated and original average number of retransmissions $1+\bar{m}$ versus the number of retransmissions with different outage threshold ϵ levels.

**Figure 3:**
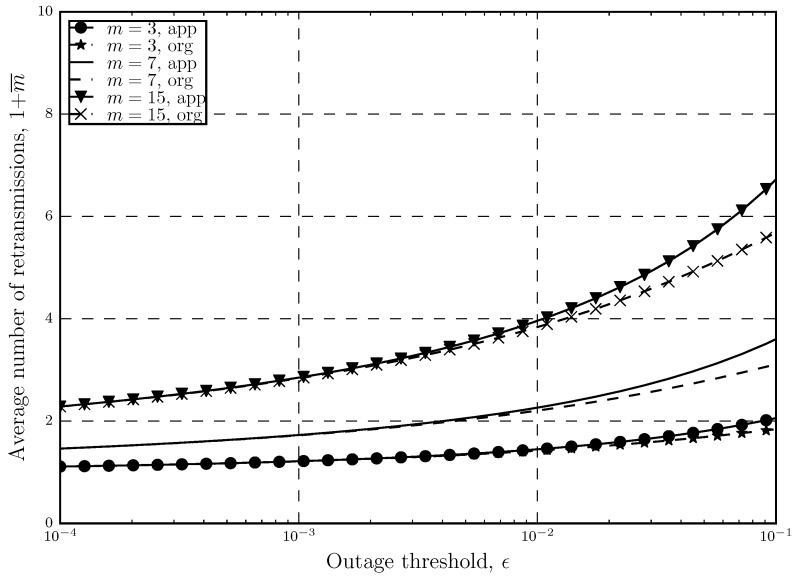
Comparison between the approximated and original average number of retransmissions $1+\bar{m}$ versus the outage threshold ϵ with different number of retransmissions.

In Figure 2 and Figure 3, we can see how (7) and ([20], Â§6) compare to each other with respect to increasing the number of retransmissions and outage threshold, respectively. From these figures, we confirm our previous claims and show the accuracy of the approximated expression. In these figures, the previously mentioned fact about ([20], Â§6) being suitable for low *m* and ϵ is also proven. It is shown that in both figures, the approximated and the original $1+\bar{m}$ are either the same or very close to each other in the mentioned areas.

**Figure 4:**
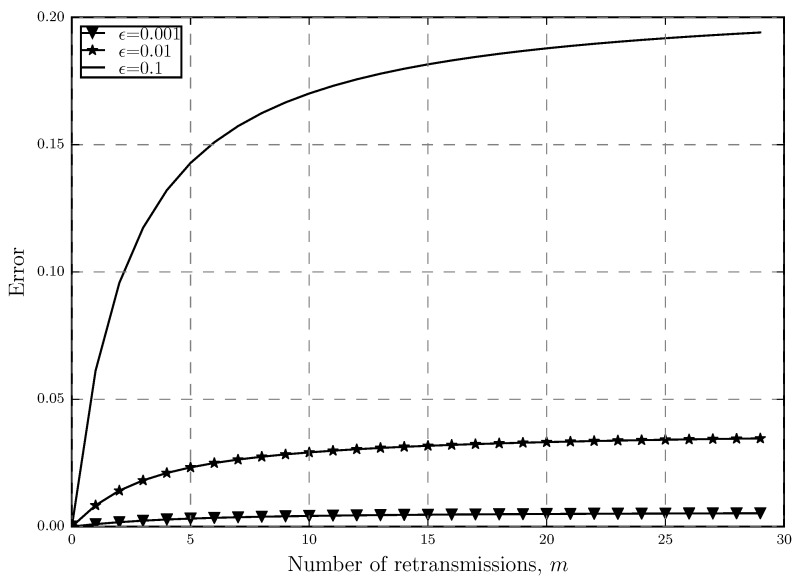
Error between the approximated and original average number of retransmissions $1+\bar{m}$ versus the number of retransmissions with different outage threshold ϵ levels.

**Figure 5:**
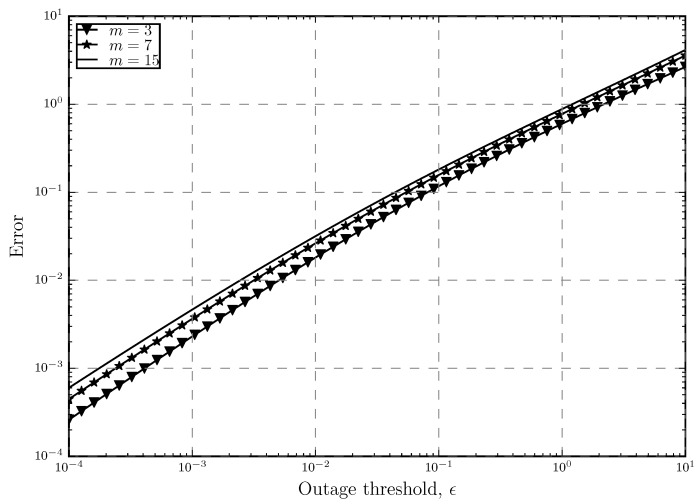
Error between the approximated and original average number of retransmissions $1+\bar{m}$ versus the number of retransmissions with different outage threshold ϵ levels.

Moving on, we analyze the error between $1+{\bar{m}}_{app}$ and $1+{\bar{m}}_{org}$ as:

(12) $$1+{\bar{m}}_{app}=\sum_{n=0}^{m}\;P_{\mathrm{out}}^{n}\approx \frac{1-P_{\mathrm{out}}^{1+m}}{1-P_{\mathrm{out}}}\approx \frac{1-\epsilon }{1-\epsilon^{\frac{1}{1+m}}}.$$

(13) $$Error=|\frac{\left(1+{\bar{m}}_{app}\right)-\left(1+{\bar{m}}_{org}\right)}{\left(1+{\bar{m}}_{org}\right)}|$$

where $\left(1+{\bar{m}}_{app}\right)$ is ([20], Equation (6)) and $\left(1+{\bar{m}}_{org}\right)$ is (7). Figure 4 shows this error as a function of number of retransmissions while Figure 5 shows how the error changes as a function of the outage threshold. We can see that as expected, the error increases when *m* or ϵ get larger. It is also shown that the increase in ϵ has a bigger impact on increasing the error. In Figure 4, when we have strict outage threshold in the network, increasing *m* does not increase the error much. However, as the outage threshold gets looser, increasing *m* results in higher error and we can see that for ϵ=0.1, error can even reach 20%. This is also true for Figure 5 where the highest error happens when both *m* and ϵ are high, further proving the point that the approximation is tight only for low *m* and ϵ.

## 4. Energy Efficiency

After the throughput optimization, we are now moving on to the energy efficiency (EE) optimization problem. This is important specially since the energy efficiency can be seen as a tool that represents the trade-off between the throughput and the total energy consumption per bit ($P_{T}$) in a network. The total energy consumption per bit in the network is itself a function of the distance dependent transmission power, total energy consumed by the radio components and bit rate [28,31,53,54]. Having this in mind, the total energy consumption per bit of this model is calculated as:

(14) $$P_{T}=\sum_{k=1}^{m+1}\frac{P_{PA}\left(k\right)+P_{T_{x}}+P_{R_{x}}}{\log(1+\beta^{*}\left(k\right))}.$$

In the above equation, $P_{PA}$ is the power consumed by the power amplifier in an one-hop communication network. This consumed power is itself a function of the drain efficiency parameter of the amplifier. This parameter is shown by δ and is δ=0.35, and hence, $P_{PA}=\frac{\beta^{*}\left(k\right)}{\delta }$. As it was also mentioned earlier, $\beta^{*}$ is the optimal SIR threshold and $\log(1+\beta^{*})$ represents the bit rate (bits/s/Hz) of the network. $P_{T_{x}}$ is the power consumed for the transmission, which is constant, and $P_{T_{x}}=97.9$ mW while $P_{R_{x}}$ is the consumed power during reception, and is $P_{R_{x}}=112.2$ mW [31]. It should be noted that both of these parameters are constant since their value depends on the current technology and also on the internal circuitry power consumption. Thus, by inserting (8) and (14) into the energy efficiency equation, which is $\frac{T}{P_{T}}$, we can express the energy efficiency of our system as:

(15) $$\mathrm{EE}=\frac{T}{P_{T}}=\frac{\log\left(1+\beta \right)\left(1-\epsilon^{\left(1+m\right)}\right)}{\left(1+\bar{m}\right)\left(\frac{\beta^{*}}{\delta }+P_{T}\right)},$$

The energy efficiency optimization problem is then:

(16) $$\begin{array}{cccc} \; & \max_{\left(\beta ,m\right)}\; & \; & \frac{\log\left(1+\beta \right)\left(1-\epsilon^{\left(1+m\right)}\right)}{\left(1+\bar{m}\right)\left(\frac{\beta^{*}}{\delta }+P_{T}\right)}\\ \; & \mathrm{subject}\;\mathrm{to}\; & \; & P_{\mathrm{out}}^{1+m}\leq \epsilon \end{array},$$

which like throughput, is also a function of SIR threshold β>0 and the number of allowed retransmissions m∈N. The energy efficiency equation in (16) is concave with respect to β if $\frac{\partial^{2}EE}{\partial \beta^{2}}<0$, and (17) obtained for $\frac{\partial^{2}EE}{\partial \beta^{2}}$ proves that this in fact is true and the energy efficiency is concave with respect to β. Energy efficiency is also concave with respect to *m*, but since the obtained expression is long and complicated, we show this concavity and the optimal throughput in the numerical results section of this paper.

(17) $$\begin{array}{c} \frac{\partial^{2}EE}{\partial \beta^{2}}=-\frac{\left(-\epsilon +1\right)\left(-\epsilon^{\frac{1}{m+1}}+1\right)\left(-\epsilon^{m+1}+1\right)}{{\left(\beta +1\right)}^{2}\left(pc+\frac{1}{\eta }{\left(-\frac{1}{k}\log\left(-\epsilon^{\frac{1}{m+1}}+1\right)\right)}^{\frac{\alpha }{2}}\right)\left(-\epsilon \left(-\epsilon^{\frac{1}{m+1}}+1\right)\left(m+1\right)-\epsilon +1\right)} \end{array}$$

## 5. Numerical Results

In this section, we present the numerical results for the previously studied optimal throughput $T^{*}$ and energy efficiency EE. It is important to mention that to obtain these results, the following arbitrary parameters where considered in our simulations. The distance between the reference receiver and sensor $r_{0}=1$ and path-loss exponent α=4; the required error rate ϵ and the density of interferers λ are the input parameters that their effects are analyzed. Moreover, based on [31], $P_{T_{x}}=97.9$ mW, $P_{R_{x}}=112.2$ mW and δ=0.35 were considered.

**Figure 6:**
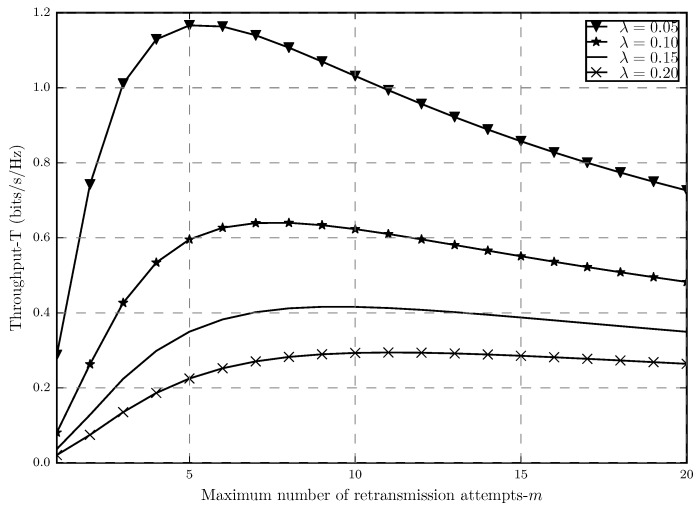
Throughput *T* versus the maximum number of allowed retransmission attempts *m* for α=4, $r_{0}=1$, ϵ=0.02 and different densities λ.

Figure 6 shows how the throughput behaves as a function of the maximum number of retransmissions in with different network densities. In this plot, we consider ϵ=0.02 in order to show how tight the previously mentioned approximation is. If Figure 6 in this paper is compared with ([20], Figure 3), we can see that the two plots are almost identical since here we have strict outage threshold in the network, which is the area where the approximation works well.

It is shown that in Figure 6, as the number of allowed retransmissions increases, the link throughput also improves until the point at which the throughput stars decreasing. This is true for different network densities as well. By increasing *m*, the system experiences a twofold effect which results in the trade-off. This effect can be explained since by increasing *m* we are allowing for also higher values of β, which also means having higher spectral efficiency in each transmission attempt (higher log(1+β)). However, by doing so, we are also increasing the outage probability since this reduces the chance of a message being correctly decoded in a single transmission attempt. In order to capture these trade-offs, we have the studied constrained optimization, whose solutions are $m^{*}$ and $\beta^{*}$, which results in the optimal throughput $T^{*}$, which represents the maximum points shown in Figure 6.

**Figure 7:**
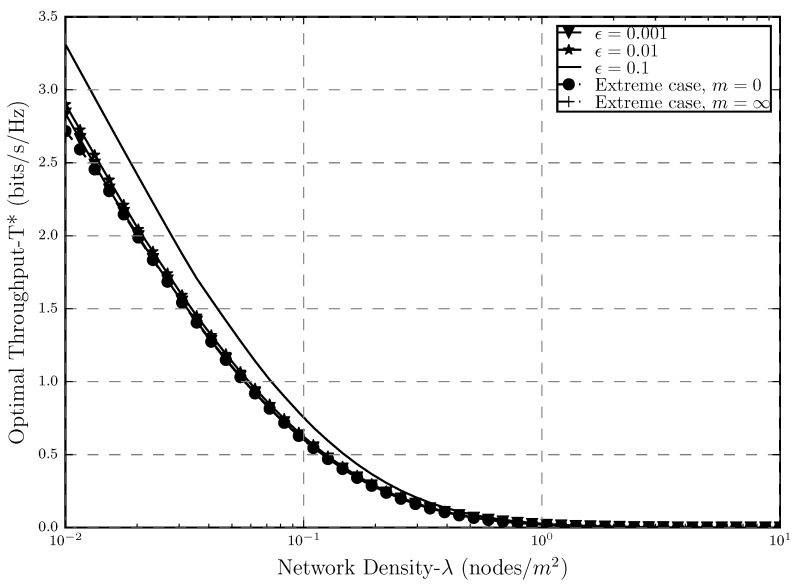
Optimal Throughput $T^{*}$ versus the density of interferers λ where α=4, $r_{0}=1$.

Figure 7 represents the optimal throughput as a function of the network density for 5 different cases. First, we can see that for the stricter outage threshold range, the optimal throughput obtained by the optimization problem in this paper is the same as the one in ([20], Figure 4), which again proves how precise the approximation is (It is important to mention that different scales for the plots are used in the two papers. In ([20], Figure 4), the linear scale is used in order to plot the optimal throughput whereas in Figure 7 in this paper, logarithmic is used, hence, the difference seen between the two plots.). Moreover, we can see how the optimal throughput decreases as λ increases. Since λ is an indicator of the number of active transmitters, i.e., source of interference in the network, it is understandable that as it increases, the throughput decreases since the unlicensed network experiences a higher level of interference from the licensed nodes.

As it was proven in Section 3.2, both extreme cases of having zero and a very large number of retransmissions will result in the same throughput. It can also be seen in Figure 7 that the optimal throughput obtained from both of these cases is also the same. One interesting notion to consider in this plot is that, when the packet error threshold is loose (ϵ=0.1), even though the number of retransmissions is not as large as m→∞, the system outperforms the other cases. In cases with limited transmissions and strict outage threshold (ϵ=0.001, ϵ=0.01), this is understandable because the stricter the error is, the worse the throughput gets, and in these cases, we are fixing the number of retransmissions to lead to the outage probability that maximizes the throughput via β. Compared to the other two extreme cases. Moreover, when m=0, we are forcing the system to have zero retransmissions in which the β would be high, and as it was shown, having higher SIR threshold would result in high outage probability as well, which will decrease the throughput. As for the m→∞, we are optimizing in terms of an error probability of 0; in other words, we are basically forcing the packet loss probability to be zero, and hence, we are losing spectral efficiency since infinite retransmissions will use a lot more spectrum resources which results in the worse throughput compared to ϵ=0.1.

In both of these extreme cases, we are taking the system degree of freedom away in terms of retransmissions and packet loss, which in the former would result in high packet loss probability and in the latter in a very high delay. On the other hand, in the proposed throughput optimization problem (4), where the outage probability and number of retransmissions are the designed variables, we are in fact relaxing the two very tight constraints that were considered in the two extreme cases, giving back the system’s degree of freedom of having a certain arbitrary level of outage while also benefiting from retransmissions in case an outage event happens. The delay of this network would be much lower than the m→0 and although the packet loss probability would not be zero, having limited retransmission would not only reduce that but would also result is the same or even higher system throughput compared to the case where m=0 which proves the benefits and importance of the optimized throughput problem studied in this paper.

**Figure 8:**
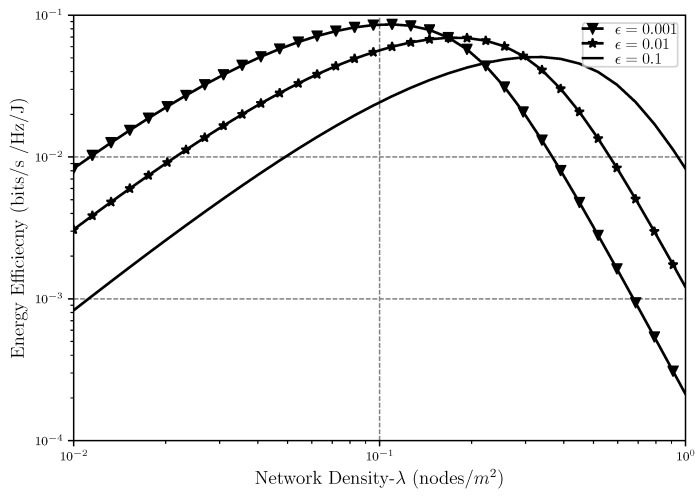
Energy efficiency EE versus the network density λ with different outage threshold ϵ levels and α=4, $r_{0}=1$.

**Figure 9:**
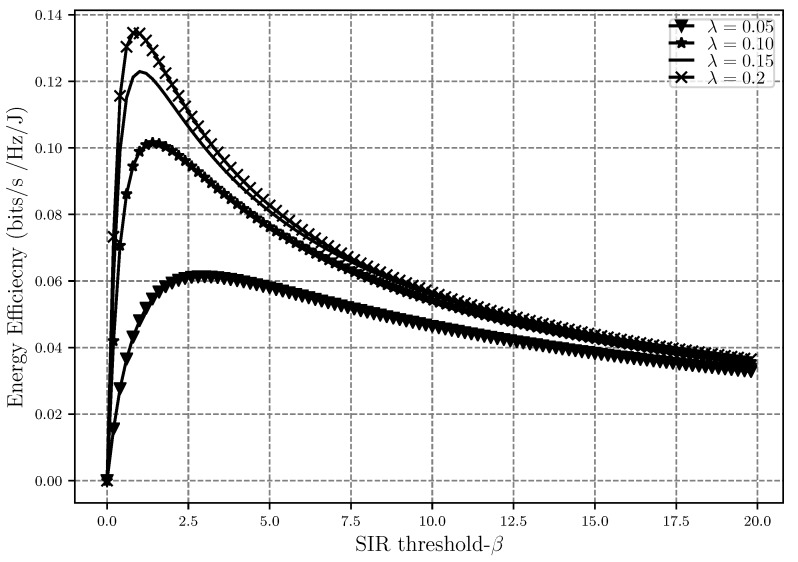
Energy efficiency EE versus the SIR threshold β with different network densities λ and α=4, $r_{0}=1$, m=5 and ϵ=0.001.

**Figure 10:**
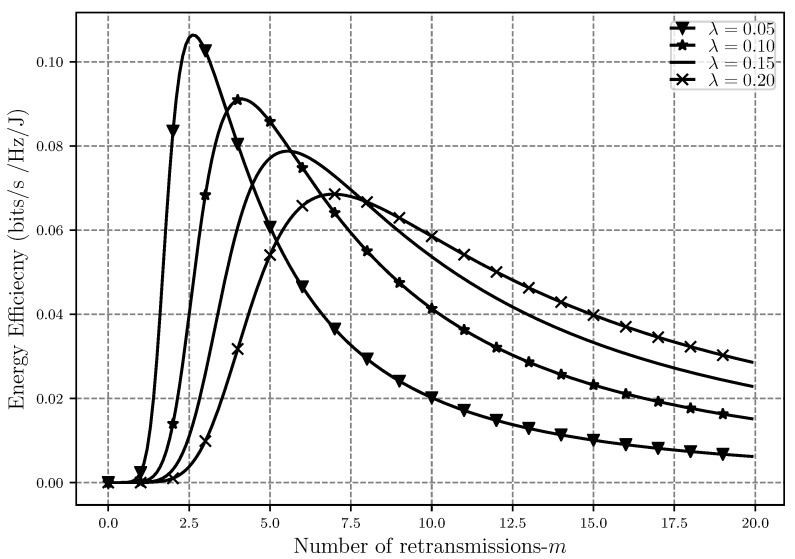
Energy efficiency EE versus the number of retransmissions *m* with different network densities λ and α=4, $r_{0}=1$ and ϵ=0.001.

Moving on to the energy efficiency analysis, EE is shown in Figure 8, Figure 9 and Figure 10 as a function of network density, SIR threshold and number of retransmissions, respectively. As can be seen in Figure 8, EE faces a decrease after some point when λ increases. When the nodes are sparsely located in the network, the level of interference is low but a lot of power is also used to transfer a message between nodes, hence EE is low. As λ gets higher, meaning that the nodes are getting closer to each other, the EE improves since less power is used for the transmission while interference is still low and the transmission is affected only by path loss. However, when the network gets very dense, the level of interference gets so high that in order to prevent outage, a lot of power should again be used for transmissions, which will cause the decrease in energy efficiency after some point in the plot.

It should be noted that when λ is low, the scenario with the loosest outage threshold has the lowest energy efficiency because the SIR in this case is lower compared to the other cases; however, when the network gets denser, the interference level rises, which would eventually decrease the throughput, and hence, the loosest outage threshold would result in the highest energy efficiency since there is more room allowed for having outage and less power is used to do the transmission in the presence of the interference. Although having a denser network also means having a higher level of interference, when there is room for higher levels of outage, less retransmissions are also needed in order to meet the reliability requirements of the network, and hence, less transmission power is used. All of these would eventually result in the system being more energy efficient in the presence of loose outage threshold in a dense network.

Figure 9 shows how EE performs as the SIR threshold gets larger while having different network densities. As β increases, it means that the throughput is also increasing, even though power consumption is also increasing at the same time. The rate at which the throughput is increasing is higher, and thus, the energy efficiency also improves during this time. This however means that the outage events are also increasing, which will decrease the throughput and eventually EE will also decrease. That is why we see the maximum point in Figure 9. Moreover, we can see that the denser the network is, the more energy efficient it is. This is due to the fact that while nodes being close to each other means higher interference, less power is used for the transmission since the distances are smaller in denser networks. This figure also shows that the energy efficiency is concave in terms of β, which was also proven in the EE optimization problem.

Moreover, the effect of increasing the number of retransmissions on EE with different network densities can be seen in Figure 10. While λ is low, lower numbers of retransmission are needed for a successful message delivery, and therefore, the energy efficiency is higher in less dense networks. As the number of retransmissions increases, we can see that while the overall EE starts to decrease, denser networks become more energy efficient as well. The reason behind this is that in networks with high λ, less power is used per each retransmission attempt to send the message since the distance between the nodes is smaller. Therefore, although the interference is higher, using less transmit power makes the network more energy efficient.

**Figure 11:**
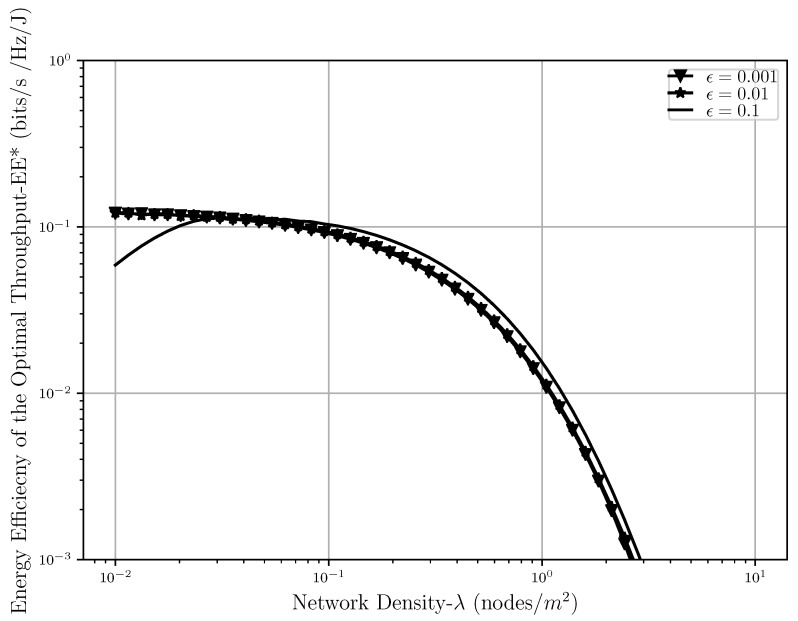
Energy efficiency of the optimal throughput $EE^{*}$ versus the number of retransmissions *m* with different network densities λ, α=4 and $r_{0}=1$.

In Figure 11 we can see the behavior of the optimal energy efficiency while λ and outage threshold level is increasing. As it was shown earlier, due to the previously explained reasons, the energy efficiency is concave with respect to λ meaning that while increasing at first, it starts decreasing after a certain density of interferers is met in the network. Hence, it is understandable that the same thing is happening in the case of optimal energy efficiency as well. It is also shown in this figure that while the scenarios when the outage threshold is somewhat strict in the network (ϵ=0.001, ϵ=0.001), $EE^{*}$ is almost the same, and the difference is negligible. However, when the outage threshold gets loose (ϵ=0.1) the optimal throughput that the system can attain is slightly higher than the other two cases. The crossing point between these cases can also be explained the same way as described in Figure 8.

## 6. Discussion and Final Remarks

In this paper, we studied the throughput and energy efficiency of a network where licensed and unlicensed nodes share the same frequency band that is used by the licensed nodes for their transmission. The interference in this model comes from the licensed network on the unlicensed users. There is also the possibility of retransmissions in case the transmitted message is not successfully decoded at the receiver. We studied the optimal throughput in a constrained optimization problem where the interferers locations are modeled using Poisson point process. We then derived the value of the optimal number of retransmissions and SIR threshold that jointly result in the optimal throughput. We showed how increasing the number of nodes and the outage threshold can decrease and increase the optimal throughput respectively and how these cases compared to the two extreme cases of having zero and infinite numbers of retransmissions in the network. It should be mentioned that the “uncoordinated” nature (ALOHA-like) of our proposed solution leads to lower throughput while using time-division or frequency-division in order to make the communication orthogonal would improve the situation. We also studied the optimal energy efficiency and how it also decreases by increasing the number of nodes and gets better by having a looser outage threshold. The energy efficiency behavior with respect to network density, SIR threshold and the number of retransmissions was also studied. In addition to the above results, we also show that the approximation used in our previous work for the average number of retransmissions is very precise and has very low error in the range. It should be noted that in this paper, we did not consider the block length since the analysis is intricate and would add to the complexity of the analysis; however, this important notion will be part of our future work.

## Figures and Tables

**Table 1:** Summary of the functions and symbols.

Symbol	Expression
$\Gamma \left[\cdot \right]$	Gamma function
$r_{i}$	Distance from the reference receiver and the ith interfering node
$g_{i}$	Channel gain
$P_{p}$	Licensed users’ transmit power
$P_{s}$	Unlicensed users’ transmit power
SIR	Signal to interference ratio
β	SIR threshold
$\beta^{*}$	Optimal SIR threshold
$\hat{\Phi }$	Poisson point process
$P_{\mathrm{out}}$	Outage probability
$P_{\mathrm{suc}}$	Probability of a successful transmission
λ	Network density (nodes/m^2^)
*T*	Link throughput
$T^{*}$	Optimal throughput
ϵ	Outage threshold
EE	Energy efficiency
α	Path loss exponent
$P_{T}$	Total energy consumption per bit
$P_{PA}$	Power amplifier consumed energy
$P_{T_{x}}$	Transmission power
$P_{R_{x}}$	Reception power
*m*	Number of retransmission attempts
$1+\bar{m}$	Average number of retransmission attempts
$1+{\bar{m}}_{e}$	Average number of retransmission attempts in the extreme cases
$m^{*}$	Maximum allowed number of retransmission attempts
δ	Drain efficiency
